# The Use of Lumbar Spine Magnetic Resonance Imaging in Eastern China: Appropriateness and Related Factors

**DOI:** 10.1371/journal.pone.0146369

**Published:** 2016-01-05

**Authors:** Liedao Yu, Xuanwei Wang, Xiangjin Lin, Yue Wang

**Affiliations:** Spine lab, Department of Orthopedic Surgery, The First Affiliated Hospital of Zhejiang University, Hangzhou, 310003, PR China; Brown University, UNITED STATES

## Abstract

Back pain is common and costly. While a general scene of back pain related practice in China remains unknown, there are signs of excessive use of lumbar spine magnetic resonance (MR). We retrospectively studied 3107 lumbar spine MRIs in Eastern China to investigate the appropriateness of lumbar spine MR use. Simple back pain is the most common chief complaint for ordering a lumbar MR study. Only 41.3% of lumbar spine MR studies identified some findings that may have potential clinical significance. Normal lumbar spine is the most common diagnosis (32.7%), followed by lumbar disc bulging and lumbar disc herniation. Walk difficulties, back injury and referred leg pain as chief complaints were associated with greater chance of detecting potentially clinically positive lumbar MR image findings, as compare with simple back pain. There was no difference in positive rates among orthopedic surgeon and specialists of other disciplines. Lumbar spine MR imaging was generally overused in Eastern China by various specialists, particularly at health assessment centers. For appropriate use of lumbar spine MR, orthopedic surgeons are no better than physicians of other disciplines. Professional training and clinical guidelines are needed to facilitate evidence-based back pain practice in China.

## Introduction

Back pain is a worldwide health problem in adults [1]. The costly diagnostic imaging and clinical interventions for back pain add considerable burden on health care systems in many countries [2]. Using standard radiologic studies, however, only a small portion of back pain patients could be precisely diagnosed with pathoanatomical findings [1]. Radiologic study, therefore, is of limited value, if past history and clinical evaluation are not suggestive of a serious underlying pathology.

Magnetic resonance (MR) imaging, which is able to provide a clear three-dimensional visualization of spinal structures, is regarded as the best non-invasive approach to detect lumbar pathologies. The most common findings on MR images perhaps are degenerative changes of the lumbar spine, such as disc degeneration, disc herniation, spinal canal stenosis, and facet joint hypertrophy. These findings, however, are common in both symptomatic and asymptomatic adults [3–6], and are weakly associated with back pain [7]. Lumbar degenerative findings, therefore, may not necessarily represent pathological changing. Rather, most of them are signs of physiological ageing. For patients with common back pain, MR have limited diagnostic and therapeutic impacts [8]. Epidemiology studies revealed that excessive imaging for back pain is associated with increased medication and spine surgery [9]. Unnecessary imaging, therefore, could be harmful to patients [10].

Yet, there is a wildly spread misconception that MR imaging is necessary to establish the cause of back pain [11, 12]. Despite the well-defined limitations, lumbar spine MR is commonly used for back pain screening and its use keeps increasing in a considerable rate [9, 13]. A number of clinical guidelines or strategies, such as red flags marking, appropriateness criteria (ACR), radiology benefits management (RBM) and clinical decision support systems [14], were developed to enhance an appropriate use of lumbar spine MR imaging in back pain practice. Despite decades of efforts to transfer scientific conclusions to clinical practice, it was estimated that one third to two thirds of spinal MR imaging may be inappropriately prescribed in the United States [15].

Little attention has been paid to back pain in China. Only recently is there an epidemiology study reported a point prevalence rate of 26% for back pain in Beijing adults [16]. Different from health care systems in Western countries, however, general practitioner and community service have yet to be developed in China. Specialists are available for direct consultation and back pain patients may refer to physicians of many disciplines. Lumbar spine MR imaging, thus, could be prescribed by various practitioners, regardless of their training background. Moreover, professional training for back pain care is scarce. Although scientific research evidenced that there is a gap between MR findings and back pain, there is a common misunderstanding among Chinese physicians that MR could identify the cause of back pain. In addition, currently there is no applicable clinical guideline for back pain practice and lumbar spine imaging in China.

While a general scene of back related practice in China remains largely unknown, there are signs of excessive use of lumbar spine MR. We retrospectively studied a large sample of lumbar spine MRIs in Eastern China to investigate the appropriateness of lumbar spine MR imaging and further to determine associated factors.

## Materials and Methods

### Study samples

The current study was conducted in Hangzhou, a typical middle-size city in China. Hangzhou is the capital city of Zhejiang Province in Eastern China, with a registered population of approximately six million. In Hangzhou, public general hospitals own all MR scanners. In Chinese health care system, public hospitals are classified as university hospitals, provincial hospitals and city hospitals, based on their size and abilities to provide medical care and education.

We aimed to include the vast majority of lumbar spine MR imaging conducted in Hangzhou city (not including satellite cities) on the January of 2013. A hospital in Hangzhou city will be included if: 1) it is a general hospital; 2) owns at least one MR scanner; 3) electronic records and images are available; 4) ethic review approved. Military hospital and specialized hospital, such as children’s hospital, women’s hospital, and cancer center, were excluded.

All lumbar spine MR imaging conducted during the defined period were included. Related data, including the patients’ age, gender, chief complaints, duration of symptoms, specialties of prescribing physician, and MR images, were extracted from Picture Archiving and Communication Systems (PACS) for analyses. The study was approved by the medical ethic board of the 1^st^ hospital of Zhejiang University. As this is only a retrospective image study, written informed consent from patient was waived and the patients’ information was anonymized and de-identified prior to any data analyses.

### Criteria for MR diagnosis

In most cases, MR reports written by local radiologists typically provided detailed descriptions of image findings but not diagnostic conclusion. Moreover, there are considerable variations of definitions in MR diagnosis of common lumbar spine disorders. In addition, there is substantial variability among radiologists of different levels. To minimize errors and standardize lumbar MR diagnoses, we specifically proposed strict MR diagnostic criteria for various lumbar spine disorders (Table 1). The criteria were proposed in such a way that the MR diagnosis could reasonably explain clinical symptoms. Degenerative findings of slight to moderate degrees, which are less likely to produce clinical symptoms, were regarded as normal. Such findings include disc degeneration (decreased signal intensity, narrowed disc space), annular tear and Modic Changes [17].

**Table 1:** MR diagnostic criteria for common lumbar spine disorders.

Diagnosis	Criteria
Disc degeneration	Significantly decreased disc signal intensity, disc space narrowing or disc bulging (without nerve root compression), as evaluated on T2W images.
Disc herniation	Disc bulging with unilateral or bilateral nerve root compression on more than one axial image of the disc. Simple disc bulging without nerve root bulging is excluded.
	Far lateral disc herniation was judged from sagittal image, with substantially narrowed intervertebral foramen and nerve root compression.
Lumbar spinal canal stenosis	Apparent central stenosis or lateral recess stenosis at two or more adjacent slices:
	(1) Central stenosis: anteroposterior diameter less than half of that at adjacent spinal level.
	(2) Lateral recess stenosis: substantial narrowed lateral recess with nerve root compression.
	(3) Foraminal stenosis: narrowed formen with significant nerve root compression, as judged from sagittal images.
Spondylolisthesis	Slip of superior vertebra relative to inferior vertebra in sagittal images.
Infection	Significant signal changes crossing over the intervertebral disc, with or without structural damage and paraspinal abscess. Both tuberculosis and bacterial infections are included.
Scoliosis	Cobb angle greater than 10 degrees in coronal images, including both idiopathic scoliosis and degenerative scoliosis.
Kyphosis	Cobb angle greater than 20 degrees in sagittal images.
Vertebral Fractures	Only fresh vertebral fractures were included, with decreased signal intensity on T1W images and increased signal intensity on T2W images. Obsolete fractures are excluded as clinically negative findings.
Spinal Tumors	Tumors at the lumbar spine and associated soft tissues, including intramedullary tumors, epidural tumors, neural tumors, bone tumors, metastasis et al.
	Fat deposition, cysts of the disc, facet joint and sacral sac, and vertebral angioma are counted as accidental findings and excluded.

A routine lumbar spine MR study conducted in Hangzhou includes 11 T1-weighted sagittal images, 11 T2-weighted sagittal images, and 9 T2-weighted axial images for the lower three lumbar intervertebral discs. There are special sequences for some cases, such as fat depression sequence and contrast sequence. All available MR images were evaluated to draw a main MR diagnosis for the imaged lumbar spine.

Three senior orthopedic surgeons, who are on average with 10 years’ experience with MRI, reviewed included lumbar spine MRIs. Before MRI evaluation, the three raters reviewed 50 sets of lumbar MRIs together to acquire a common sense of the proposed diagnostic criteria. The MRI review was performed in a PACS workstation and the raters were blinded to the patient’s history and physical examinations.

### Definition of potentially clinically positive diagnosis

Based on potential clinical relevance, MR diagnosis was classified as potentially clinically positive or clinically negative. MR diagnoses of systematic lumbar spine diseases or neurologic compression which may need clinical interventions are defined as potentially clinically positive diagnosis, including lumbar disc herniation, lumbar spinal canal stenosis, spondylolisthesis, spinal tumor, spinal infection, fresh vertebral fracture, and spinal deformity (scoliosis or kyphosis). Findings that are common among both healthy individuals and back pain patients, which deserve little or no clinical attention, were classified as clinically negative diagnosis, including disc degeneration, high intensity zone in the disc, obsolete vertebral fractures, fat deposition in the vertebral body, Modic Changes, facet joint degeneration, and cysts on the disc, facet joint or sacral canal.

A MR study was regarded as appropriate if there are potentially clinically positive findings on MR images. Otherwise, it was considered to be inappropriate.

### Statistical analysis

Data were analyzed using STATA (Version 12.0, StataCorp LP, USA). Descriptive statistics were used to compare findings among hospitals, departments, and physicians of different specialties. Multivariable logistic regressions were used to determine the associations between potentially clinically positive findings and related factors, and the associations between positive lumbar MR findings and the duration of a specific chief complaint.

## Results

Except for three relatively small hospitals, all general hospitals in Hangzhou city which own a MR scanner were included in the current study. As a result, we studied 10 leading hospitals of various ranks in Hangzhou, including 3 hospitals affiliated to Zhejiang University, 3 provincial hospitals and 4 city hospitals. For the three excluded hospitals, electronic MR images database was not available in two and ethic permission was not obtained in another.

From January 1^st^ to January 31^st^ of 2013, there were 3107 patients (1369 male and 1738 female patients, age 52.73±16.14 years, range 3 to 100 years) underwent lumbar MR imaging at the included 10 hospitals. Among them, 1406 (45.3%) cases were conducted at hospitals affiliated to Zhejiang University, 752 (24.2%) cases at provincial hospitals, and 949 (30.5%) cases at city hospitals. There are 2513 outpatient cases (81.1%) and 285 hospitalized patients (18.9%).

Of all the lumbar spine MR studies, 64.1% were ordered by orthopedic surgeons, 21.7% by various specialists of internal medicine, 7.2% by neurologists and neurosurgeons, and the remaining 7.0% by the practitioners at health assessment centers which specifically provide routine health evaluation service.

### The rate of potentially clinically positive diagnosis

**Fig 1:**
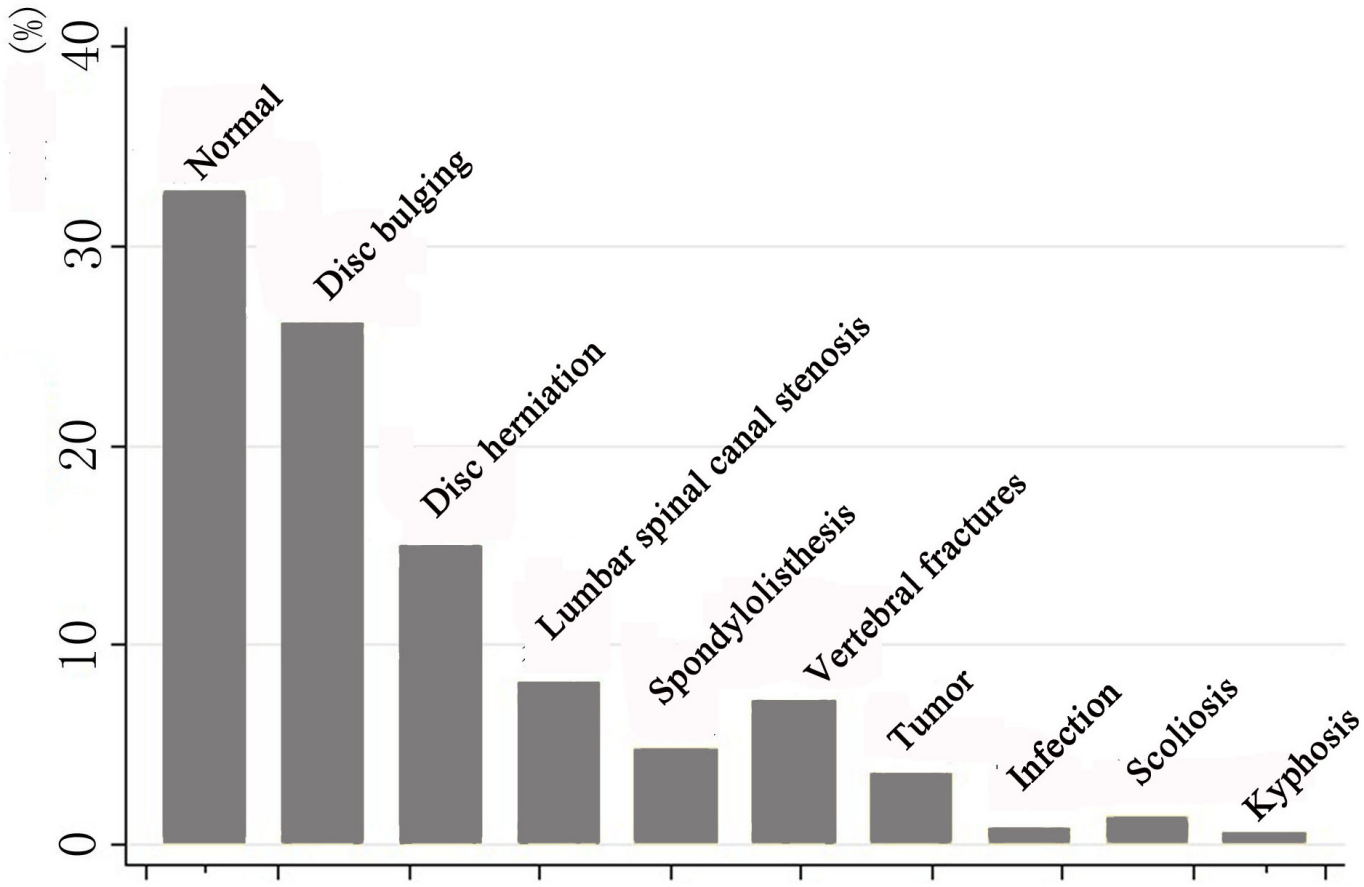
MR diagnosis evaluated by orthopedic surgeons for 3107 lumbar spine MR studies.

Using our definition, only 41.3% of all lumbar spine MR studies were considered as potentially clinically positive diagnosis. Findings of the remaining 58.3% lumbar spine MRIs were regarded as clinically negative. Normal lumbar spine is the most common diagnosis (32.7%) on lumbar spine MRIs, followed by lumbar disc bulging (26.2%) and lumbar disc herniation (15.0%) (Fig 1).

### Chief complaints for ordering a lumbar MR study

**Fig 2:**
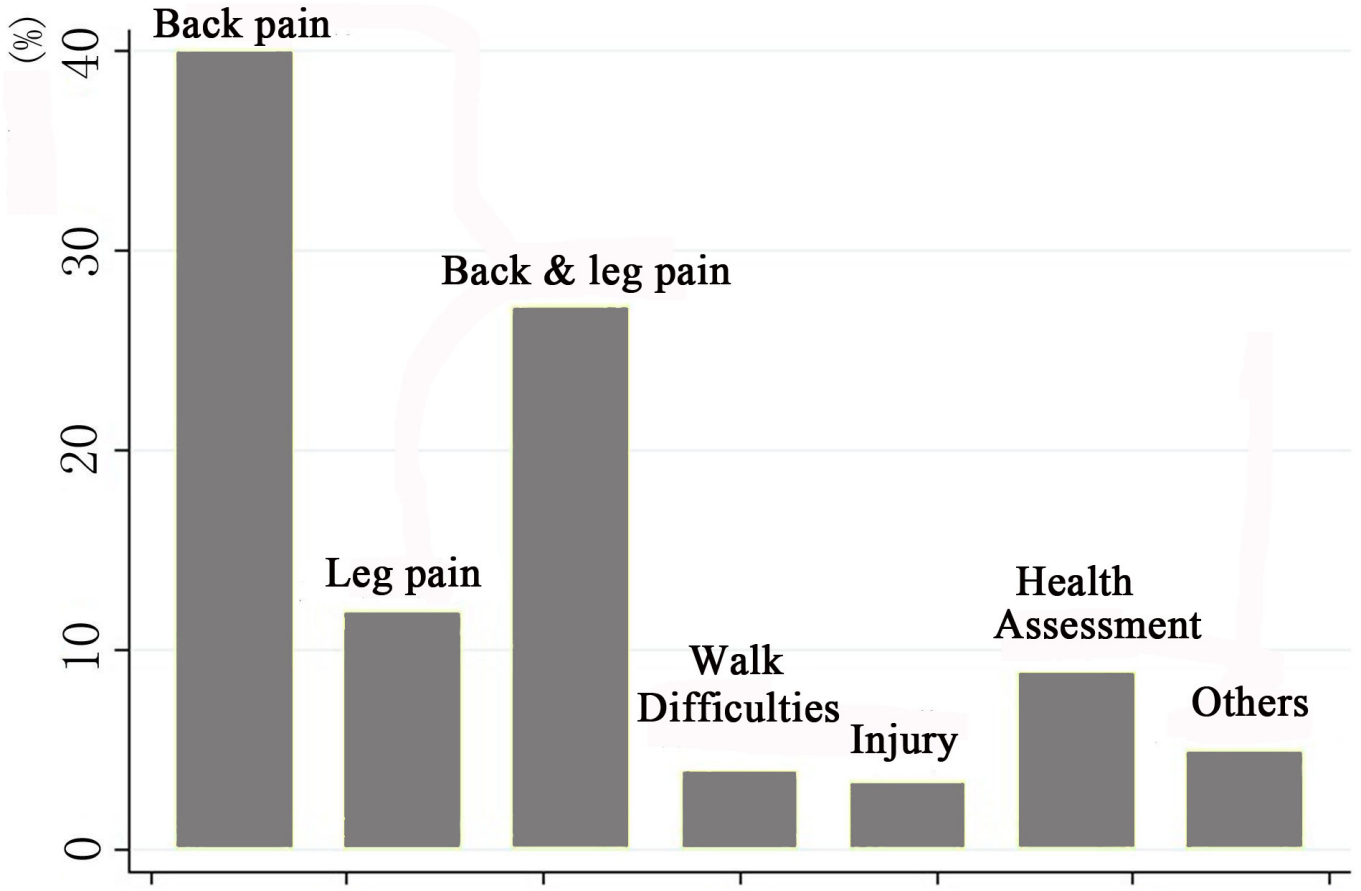
The constitution of chief complaints for ordering a lumbar spine MR study (N = 3107).

Simple back pain is the most common chief complaint for ordering a lumbar MR study (40.0%), followed by back pain with radiating leg pain (27.2%) and simple leg pain (11.8%). The constitution of chief complaints is presented in Fig 2.

### Factors associated with the rate of potentially clinically positive diagnosis

The rates of potentially clinically positive diagnosis in relation to patient’s age, gender, chief complaint, hospital rank and prescribing physician are presented in Table 2. A trend of greater age associated with higher rate of potentially clinically positive diagnosis was clear.

**Table 2:** Percentage of potentially clinically positive diagnosis in relation to studied factors.

	Sample size (N)	Rate of potentially clinically positive diagnosis (%)
**Patient’s Age**		
<20	31	48.4
20–30	246	23.6
30–40	481	20.4
40–50	654	29.5
50–60	730	39.5
60–70	516	57.2
70–80	276	71.7
>80	173	79.2
**Patient’s gender**		
Female	1 738	40.5
Male	1 369	42.3
**Chief complaint**^§^		
Back pain	998	34.1
Radiating leg pain	295	44.8
Back and leg pain	679	42.7
Walking difficulties*	98	71.4
Back injury	84	67.9
Health assessment	220	19.6
Others**	123	53.7
**Hospital rank**		
University	1 406	39.1
Provincial	752	38.3
City	949	46.7
**Prescribing physician**^§§^		
Orthopedic surgeon	1 914	43.2
Neurologist and neurosurgeon	185	44.0
Specialist of internal medicine	647	41.9
Practitioner at health assessment center	210	16.2

^§^N = 2497

^§§^N = 2985, due to missing data.

*Claudication, difficult or unable to walk;

**Mainly include those with a history of lumbar spine disorders, tumor, infection or surgery and need a MR follow-up study.

A multiple variable regression model was used to explore the determinants for the rate of potentially clinically positive diagnosis (Table 3). Male patients were statistically significantly associated with greater rate of potentially clinically positive MR findings, as compared with female patients (OR = 1.22, P = 0.03). Chief complaints were important determinants for potentially clinically positive MR findings. Except for health assessment, leg pain, back and leg pain, walk difficulties, and back injury as chief complaints were statistically significantly associated with greater possibility of detecting potentially clinically positive lumbar MR findings, as compare to simple back pain (Table 3).

**Table 3:** Associations between presence of clinically significant MR findings and related factors: results from multiple variable regression analysis*.

Variable	OR	95% CI	P
**Age**	1.05	[1.04, 1.05]	<0.001
**Gender**			
Female	1.00		
Male	1.22	[1.02, 1.46]	0.030
**Chief complaint**			
Back pain	1.00		
Leg pain	1.32	[0.96, 1.79]	0.083
Back and leg pain	1.42	[1.14, 1.77]	0.002
Walking difficulties	3.92	[2.32, 6.62]	<0.001
Back injury	4.31	[2.59, 7.18]	<0.001
Health assessment	0.91	[0.55, 1.51]	0.717
Others	2.04	[1.36, 3.08]	0.001
**Hospital rank**			
University	1.00		
Provincial	0.72	[0.57, 0.90]	0.005
City	0.93	[0.72, 1.19]	0.551
**Prescribing physician**			
Orthopedic surgeon	1.00		
Neurologist and neurosurgeon	1.03	[0.71, 1.50]	0.865
Specialist of internal medicine	0.84	[0.67, 1.05]	0.131
Practitioner at health assessment center	0.36	[0.21, 0.60]	<0.001

*: Due to data missing, N = 2395.

Provincial hospitals, but not city hospitals, were statistically associated with lower rate of positive MR findings, as compared with university hospitals. Practitioners at health assessment centers had significantly lower rate of potentially clinically positive findings than orthopedic surgeons (OR = 0.36, P<0.001). There was no statistically significant difference among the rates of potentially clinically positive findings among orthopedic surgeon, neurologist/neurosurgeon, and specialist of internal medicine, after adjusting for other confounding factors.

### Duration of chief complaint and potentially clinically positive diagnosis

The duration of simple back pain, leg pain, back and back pain, and walk difficulties were not associated with greater rate of potentially clinically positive lumbar spine MR diagnosis, adjusting for age and gender (Table 4).

**Table 4:** Associations between the presence of potentially clinically positive MR findings and duration of chief complaints, adjusting for age and gender.

Chief complaint	Back pain (N = 998)		Leg pain (N = 295)		Back & leg pain (N = 679)		Walking difficulties (N = 97)	
**Duration**	**OR**	**P**	**OR**	**P**	**OR**	**P**	**OR**	**P**
**< 1 month**	1.00		1.00		1.00		1.00	
**1–3 months**	0.86	0.54	0.75	0.42	1.25	0.38	1.01	0.99
**3–12 months**	0.84	0.46	0.71	0.32	1.53	0.09	0.99	0.99
**>12 months**	0.74	0.07	0.58	0.09	0.88	0.49	0.45	0.12

## Discussion

For the first time, the appropriateness of lumbar spine MR use in China was investigated. The current study revealed that most lumbar spine MR imaging did not identify any finding that may have potential clinical significance, suggesting that lumbar spine MR imaging was considerably overused in Eastern China. In particular, lumbar MR was abused at health assessment centers, as merely a rather small percentage of lumbar MR imaging proscribed there detected findings may have potential clinical significance. Moreover, the rates of potentially clinically positive findings on lumbar spine MRIs were similar among specialists of various disciplines, suggesting that there may be a general insufficiency in back pain related training for physicians in China.

It should be noted that the identified positive rate of 41.3% may represent a maximal rate of appropriateness of lumbar spine MR use. Occasionally, clinical symptoms and history strongly indicated for a lumbar MR study but as a result identified no significant findings. Therefore, MR imaging with negative findings does not fully represent inappropriate use of MR as it may have ruled out suspicious pathologies. To a larger degree, however, MR may be inappropriately used for those without any positive finding on MRIs. On the other hand, the identified positive lumbar MR findings may not necessarily mean to have clinical significance, as MR is not able to reliably differentiate symptomatic from asymptomatic spinal image abnormalities [4]. Some of them are incidental findings that are not responsible for the current complaints [18]. For example, disc herniation and lumbar spine canal stenosis are common findings on asymptomatic volunteers and thus, may not be able to explain the current back pain [18]. In addition, MR imaging does not influence or change clinical management, even if clinical symptoms could be attributable to the identified MR findings. As such, the true appropriateness rate for lumbar spine MR use may be much lower than that observed in the current study.

As an important component of back pain management, the appropriateness of lumbar spine MR utilization has been studied in developed countries using ACR criteria [12], expert opinion [19], or other criteria derived from clinical guidelines [20, 21]. The reported rates of appropriateness of lumbar MR use ranged considerably from 12% to 56.7%. Different from previous studies, we used image findings, as assessed by experienced orthopedic surgeons, to estimate the appropriateness of lumbar MR use. As MR diagnosis criteria for lumbar disorders vary, we specifically proposed a restrict MR diagnosis criterion to minimize errors from evaluators’ conceptual differences on MR findings. This diagnosis protocol counts only severe image findings that deserve further clinical attentions. Mild and moderate degenerative pathologies, such as disc degeneration and canal stenosis, which are less likely associated with current pain and thus, were regarded as accidental findings did not cause clinical symptoms. Although the criteria and cut-off rationale remain controversial, we think it is reasonable for appropriateness evaluation, given the fact that a severe image finding is typically regarded as an indicator for clinical intervention.

There are several reasons that may explain the relatively high rate of inappropriate use of lumbar spine MR imaging in China. It is well-known that explicit guidelines to support physician’s decision making could improve clinical practice and outcomes [22]. Yet, the managements of back pain and related problems are inadequately addressed in the developing country of China. Applicable clinical guidelines for back pain screening and appropriate use of lumbar spine MR imaging are currently absent in China. As a result, back pain practice largely relies on physicians’ clinical experience. On the other hand, back pain education in China is inadequate. Resident training or post-graduate education was absent for most Chinese physicians in the past half century. As there is no general practitioner for back pain screening, most back pain patients directly visit orthopedic surgeons. One may expect that the rate of appropriate use of lumbar MR would be higher with orthopedic consultation than that with general practitioners, as did in many countries. Yet, the rate of potentially clinically positive findings was low, despite 65% of lumbar spine MR imaging was ordered by orthopedic surgeons. The appropriateness rates were not different between orthopedic surgeons and other physicians, suggesting that the excessive use of lumbar spine MR is a holistic problem and professional back pain training is generally insufficient for most physicians.

Other possible reasons for inappropriate use of lumbar spine MR include medical liability fears, meeting patient’s demand, and economic motivation. Reportedly many practitioners order a lumbar MR study to meet the patients’ expectations about MR tests [23]. Another unexpected finding is that the rate of potentially clinically positive MR findings was only 16% at health assessment centers. Such department, however, is highly economically motivated. Lumbar spine MR imaging is prescribed on a self-service basis, or recommended by practitioners there without any screening.

Although there is no radiation exposure, MR imaging may do more harms than good [10]. Even among patients with back pain, MRI findings may be misleading. Incidental findings may lead to over diagnosis and over medication. In addition to the high costs of MR imaging and consequent medication, increased use of lumbar MR imaging is associated with higher rates of spine surgery without better outcomes [9]. Moreover, label patients with a MR diagnosis may result in fear, anxiety and depression in the patients and dependence on medical care [24]. A lumbar MR study, therefore, should be saved for those who potentially have underlying serious systemic problems and should not be used as a routine for health assessment. Targeted use of lumbar spine MR should be emphasized as it can reduce patients’ health expanse, shorten waiting time, and promote the reasonable allocation of medical resources [25, 26].

The current study confirmed that walk difficulties and back injury are better indicators for a lumbar MR study, as relative to simple back pain. This is consistent with previous studies that back pain without referred leg pain is a risk factor for inappropriate prescription of lumbar spine MR [20, 21]. Opposing to common view, however, increased duration of back pain, leg pain or walk problems was not associated with greater chance of detecting potentially clinically significant findings on MR images. Chronic low back pain, a condition without radiculopathy or anatomical abnormalities clearly responsible for the pain [1], could explain the identified association between back pain duration and negative MR findings. Referred leg pain and difficulties in walking are clear signs of nerve root compression or stenosis [1] and thus, the presence but not duration is related to significant MR findings.

The current study is a retrospective investigation of lumbar spine MR use in a typical city of Eastern China. As the use of lumbar spine MR may vary in different geographic regions, it may not be able to represent that in whole China. Although the diagnostic criterion proposed is restrict, we did not take clinical history or physical examinations into consideration. While most leading hospitals in a typical city were studied and the study sample is large, we had experienced orthopedic surgeons reviewed all MR images to have a precise image diagnosis. Nevertheless, the present study provides a preliminary overview of the current status of back pain practice in China.

In summary, there is considerable overuse use of lumbar spine MR imaging by various specialists in Eastern China. Lumbar spine MR should not be routinely used for the purpose of back pain screening and health assessment. Professional training on back pain screening and clinical guidelines for the management of back related conditions are needed to promote effective allocation of medical resources and to facilitate evidence-based practice in China.
